# Discrepant Bleeding Patterns in a Case of Simultaneous Injuries: Hemodynamic Consequences of Bilateral Carotid Artery Transection

**DOI:** 10.7759/cureus.88377

**Published:** 2025-07-20

**Authors:** Ikuto Takeuchi, Motoo Yoshimiya, Atsushi Ueda, Yu Kakimoto

**Affiliations:** 1 Department of Forensic Medicine, Tokai University School of Medicine, Isehara, JPN

**Keywords:** carotid artery transection, forensic pathology, hemodynamic shunting, multi-trauma injury timing, wound vitality

## Abstract

In forensic practice, differences in hemorrhage volume among multiple injuries are often used to estimate the timing of trauma. We report a rare case in which multiple injuries sustained simultaneously exhibited marked discrepancies in hemorrhagic presentation. A man in his 30s was found deceased at the scene of a single-vehicle accident, in which his car had collided with a barrier. Forensic autopsy revealed complete bilateral transection of the common carotid arteries, cervical spinal cord transection, and cervical spine fractures. Additional injuries included a liver laceration and a lower leg contusion, both of which showed minimal associated hemorrhage. The bilateral carotid artery injury was determined to be the immediate cause of death. Based on hemodynamic principles, including Hagen-Poiseuille’s law, we hypothesize that the abrupt reduction in vascular resistance at the transected carotid stumps formed a low-resistance, short-circuit pathway, diverting the majority of cardiac output toward the open carotid arteries. This resulted in rapid exsanguination and minimal bleeding at other injury sites. This case underscores the importance of accounting for hemodynamic factors when evaluating wound vitality, as exclusive reliance on hemorrhage volume may lead to misinterpretation of injury timing or cause of death, particularly in complex trauma cases.

## Introduction

In forensic practice, differences in the amount of hemorrhage among multiple injuries are often interpreted as important findings that suggest the timing of trauma. It is widely recognized that wounds inflicted after cardiac arrest do not exhibit vital reactions, such as hemorrhage, inflammation, or tissue swelling [1]. On the other hand, in cases of severe trauma, it is not uncommon for multiple organs to sustain fatal injuries simultaneously, and, in general, the severity of each injury is assessed using the Abbreviated Injury Scale [2]. However, to our knowledge, there are no reports investigating the circulatory effects that these injuries exert on one another, and it remains unclear how the anatomical relationships of the organs and changes in hemodynamics might create differences in hemorrhagic findings when such injuries occur simultaneously.

Additionally, it is known that bleeding associated with carotid artery injury can lead to a rapidly fatal course due to its active nature [3]. While there are several reports describing cases of carotid artery dissection or unilateral carotid artery transection [4,5], to our knowledge, there are no reports documenting the clinical course or treatment of complete bilateral carotid artery transection. In adults, cardiac output is generally estimated at approximately 4-6 L per minute, while cerebral blood flow accounts for about 700-800 mL per minute [6,7]. Moreover, detailed examinations of postmortem hemodynamic estimation and discrepancies in hemorrhage volume among wounds in such cases are extremely limited.

Against this background, we present a case demonstrating a unique finding of rapid circulatory collapse caused by bilateral carotid artery transection, along with minimal hemorrhage observed at other sites of simultaneous injury. By discussing the hemodynamic mechanisms underlying this phenomenon, we aim to provide perspectives that may contribute to cause-of-death estimation and wound assessment in cases of multiple traumatic injuries.

## Case presentation

A man in his 30s was found deceased at the scene of a single-vehicle accident in which his car had collided with a barrier at the end of a T-intersection. The front of the vehicle was heavily damaged, and a traffic sign pole was found to have penetrated the driver’s compartment. Based on the body position and external injuries, the pole was presumed to have impaled the driver’s neck. Emergency medical personnel judged the case to be non-survivable, and the body was referred for forensic autopsy.

**Figure 1:**
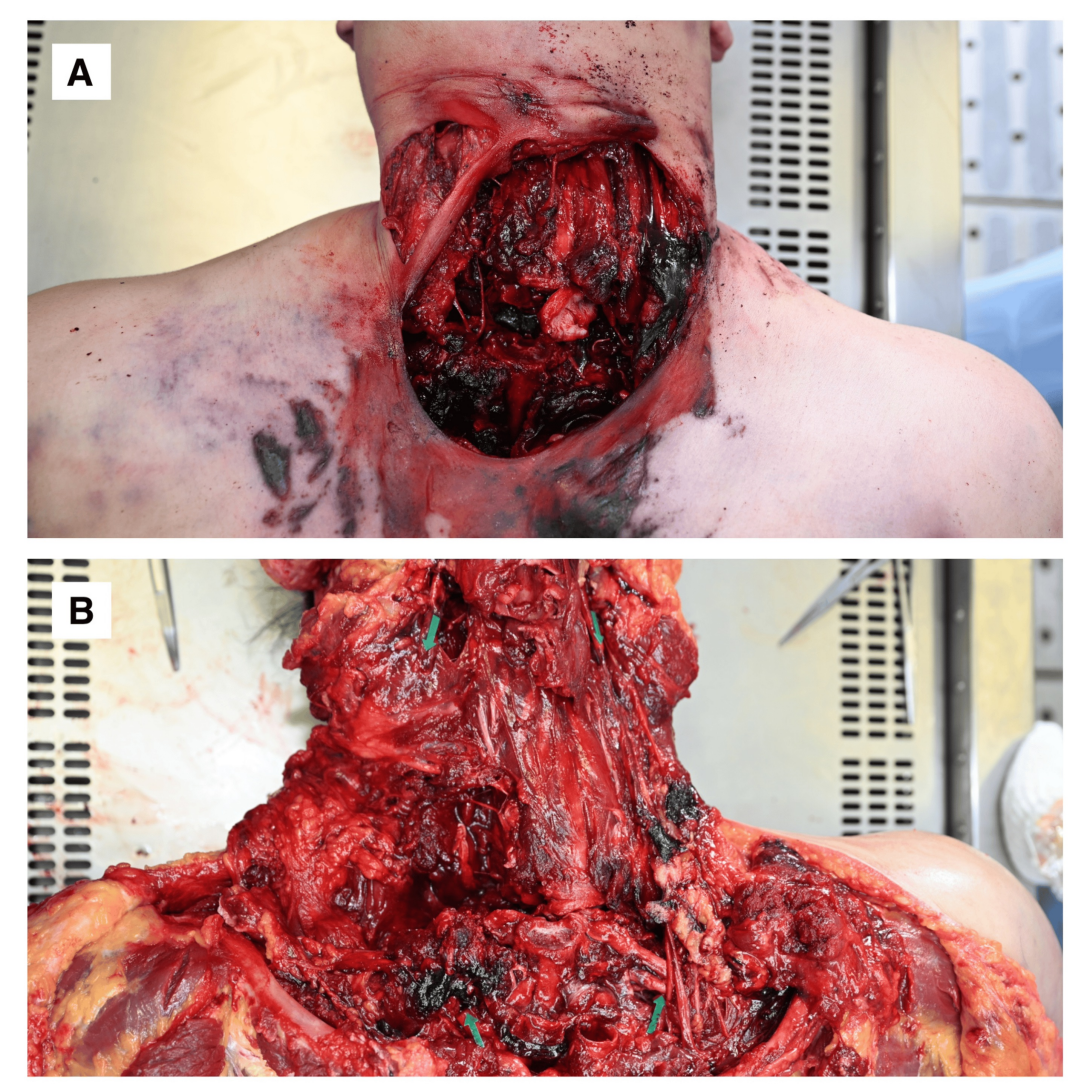
External and Internal Findings of Common Carotid Artery Transection (A) External view of the neck showing injury location. (B) Autopsy photograph demonstrating complete transection of both common carotid arteries; transected arterial stumps are indicated by arrows. Due to extensive disruption and fragmentation of the neck region, further dissection and detailed photographic documentation of the carotid arteries were not feasible.

Postmortem examination revealed complete transection of both common carotid arteries (CCAs) (Figure 1), cervical spine fractures, and transection of the cervical spinal cord.

Due to the extensive disruption and fragmentation of the neck region, further dissection and detailed photographic documentation of the carotid arteries were not feasible.

**Figure 2:**
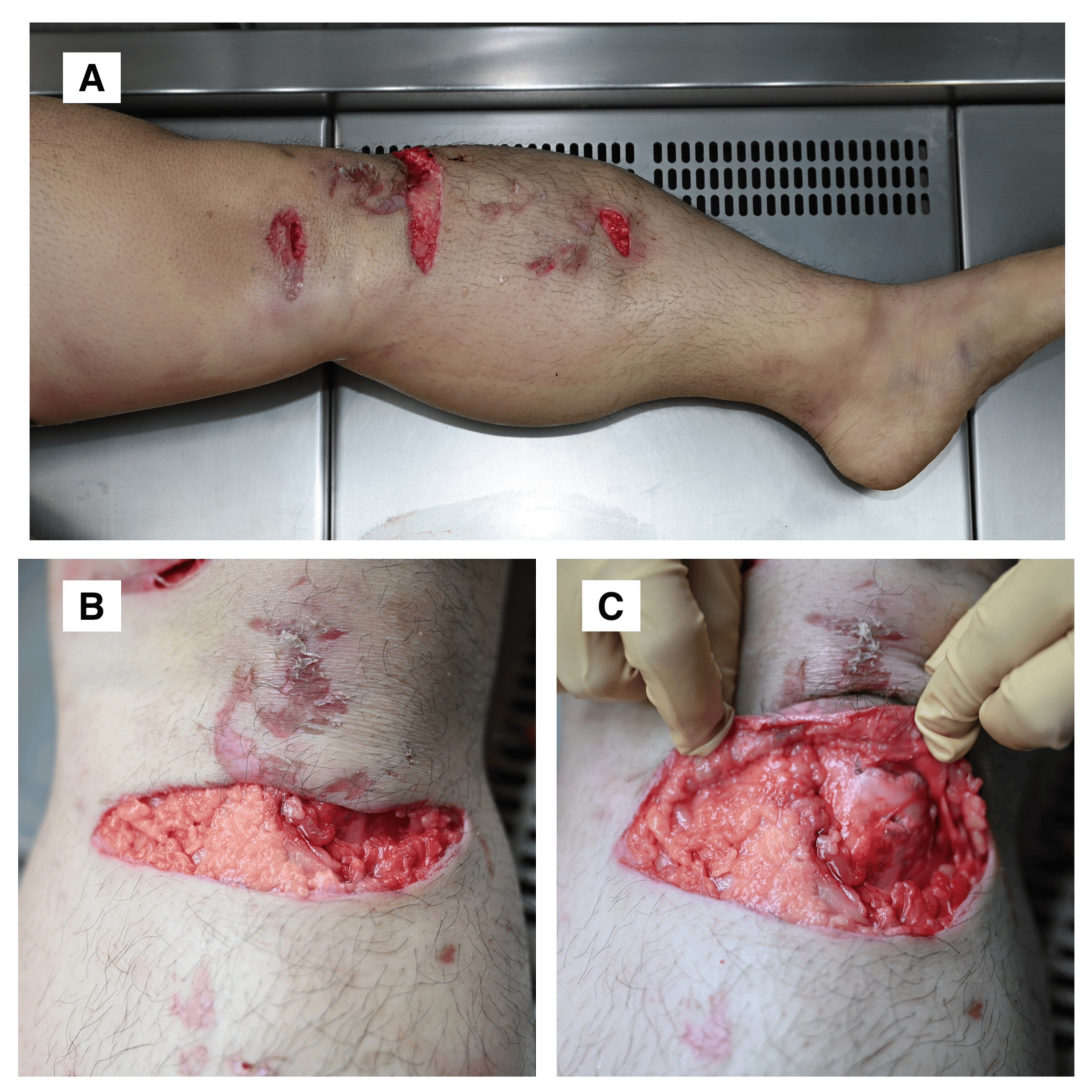
Lower Leg Laceration With Minimal Subcutaneous Hemorrhage (A) Overview of the entire lower leg showing the injury site. (B) Close-up view of the laceration site on the lower leg. (C) Subcutaneous dissection revealing minimal hematoma formation beneath the laceration.

A lower leg laceration was also noted, with minimal associated subcutaneous hematoma (Figures 2A-2C).

**Figure 3:**
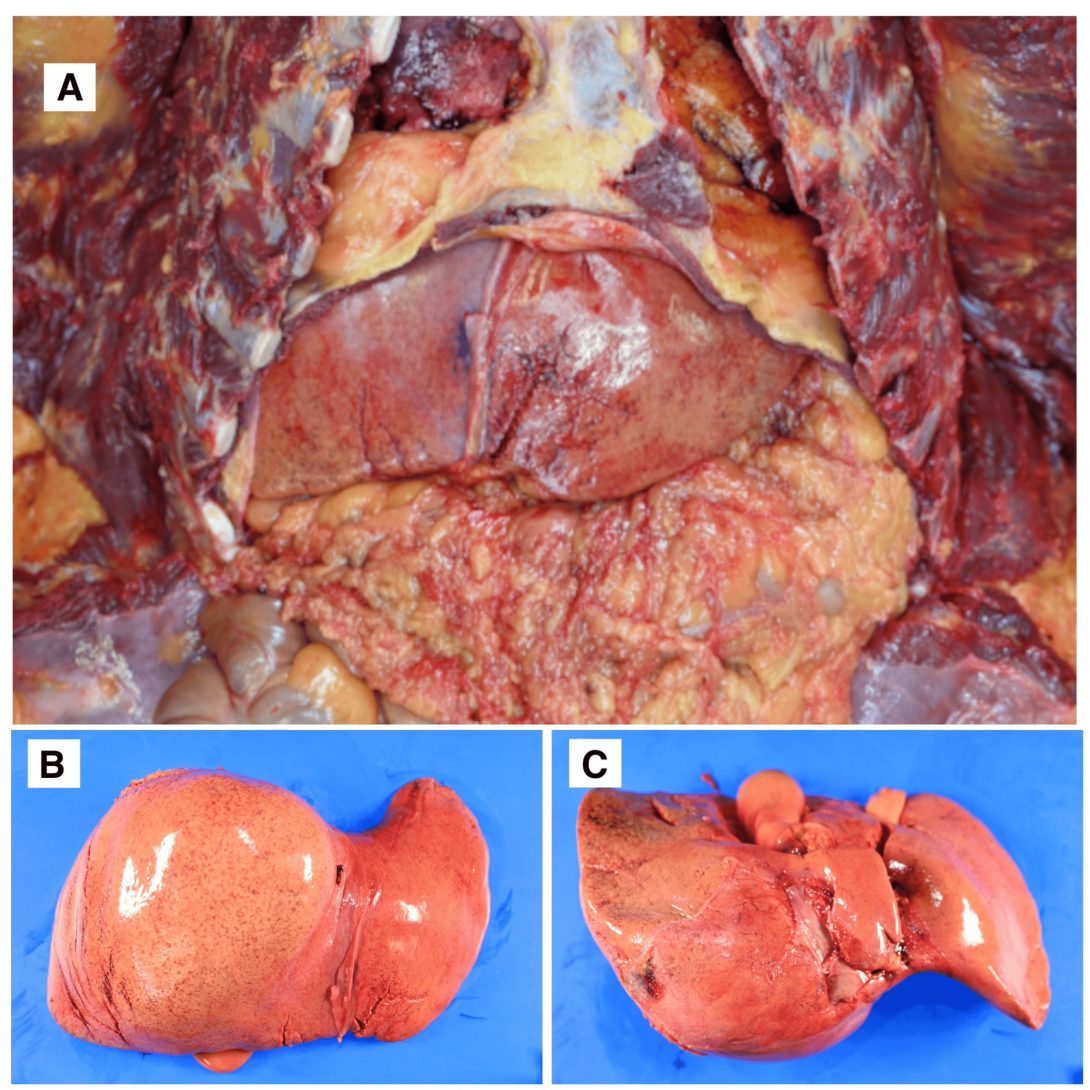
Liver Laceration With Minimal Hemorrhage (A) Liver appearance at initial abdominal opening. (B) Anterior view of the liver showing laceration with minimal hemorrhage. (C) Posterior view of the liver showing limited hematoma formation.

Although a liver laceration was identified, the associated hemorrhage was minimal and insufficient for collection (Figures 3A-3C).

Police investigation confirmed that all injuries had been sustained in the crash. While the bilateral carotid artery injury was determined to be the immediate cause of death, the minimal hemorrhage at the liver and leg injury sites warranted further forensic analysis.

## Discussion

In this case, fatal trauma resulted from bilateral transection of the CCAs. A notable feature was the markedly limited hemorrhage observed at other injury sites (the liver and lower leg), despite the severity of the cervical trauma. While fatal exsanguination following carotid artery injury is well-documented [3], reports of simultaneous multiple injuries with markedly different hemorrhage volumes are rare. Understanding the hemodynamic basis of such discrepancies is essential for accurate forensic interpretation.

Cardiac output in adults is generally estimated at 4-6 L per minute, with cerebral blood flow accounting for approximately 700-800 mL/min [6,7]. Under normal conditions, blood is distributed relatively evenly throughout the body. However, transection of the carotid arteries causes a sudden drop in vascular resistance in the cerebral circulation, converting the severed stumps into low-resistance, short-distance outflow tracts. This results in the preferential diversion of blood flow into these open ends.

According to Hagen-Poiseuille’s law, blood flow (Q) is proportional to the fourth power of the vessel radius and inversely proportional to its length [8]. This principle is commonly applied in clinical settings, such as infusion flow rates through IV catheters, where both internal diameter and catheter length influence flow dynamics [9,10]. The average length of the CCA is approximately 13.6 cm (right) and 12.4 cm (left), and the internal carotid artery is about 8.5 cm [11]. In the present case, transection occurred near the mid-portion of the CCA, reducing the effective vessel length to one-third or even one-fifth. Such a reduction greatly lowers resistance, increasing flow through the open stump. If cerebral blood flow increased fivefold, it could theoretically match total cardiac output, allowing the carotids to act as the dominant outflow tracts. This preferential shunting likely deprived the descending aorta of effective perfusion, thereby explaining the minimal hemorrhage in other injured regions.

Due to the severity of trauma and the lack of retained blood at the scene or within body cavities, accurate estimation of blood loss was not feasible. However, based on anatomical findings and known hemodynamic principles, death likely occurred within minutes. Police investigation excluded postmortem trauma. The liver injury corresponded to abdominal compression from the seatbelt [12], and the lower leg contusion was consistent with impact against the dashboard [13].

This case offers important forensic insights. In evaluating multiple injuries, distinguishing between ante- and post-mortem trauma is critical. Vitality indicators, such as hemorrhage, must be interpreted within a broader physiological and contextual framework. As shown here, relying solely on bleeding patterns may lead to misinterpretation under altered hemodynamic states. A comprehensive assessment, integrating anatomical findings, physiological reasoning, and scene information, is essential in trauma chronology and cause-of-death analysis [14,15].

## Conclusions

This case demonstrates that bilateral carotid artery transection can create a hemodynamic shunt that redirects most cardiac output into the severed vessels, resulting in minimal hemorrhage at other injury sites. Application of Poiseuille’s law explains this physiological phenomenon and underscores the need to interpret wound bleeding in context. The findings highlight that bleeding volume alone may not indicate injury timing, especially in complex trauma. Forensic evaluations must integrate anatomical, physiological, and situational factors to avoid misinterpretation of trauma chronology and cause of death.
